# Evaluating breed and seasonal effects on milk yield, composition, and cheesemaking ability of 2 Italian dairy sheep breeds

**DOI:** 10.3168/jdsc.2025-1006

**Published:** 2026-05-15

**Authors:** Silvia Graziani, Simone Callegaro, Claudio Cipolat-Gotet, Maria Chiara Fabbri, Giorgia Stocco, Lina Fernanda Pulido-Rodríguez, Davide Valoppi, Irene Tedeschi, Francesco Sirtori, Bianca Castiglioni, Silverio Grande, Christian Maltecca, Riccardo Bozzi, Stefano Biffani, Francesco Tiezzi

**Affiliations:** 1 Department of Agriculture, Food, Environment and Forestry, University of Florence, 50144 Florence, Italy; 2 Department of Veterinary Sciences, University of Parma, 43126 Parma, Italy; 3 Institute of Agricultural Biology and Biotechnology, National Research Council, 20133 Milan, Italy; 4 Ufficio Studi, Associazione Nazionale della Pastorizia, 00187 Rome, Italy; 5 Animal Science Department, North Carolina State University, Raleigh, NC 27695

## Abstract

This study aimed to characterize milk yield, composition, and cheesemaking traits of Comisana and Massese ewes managed under uniform conditions and to evaluate the effects of breed, season, and their interaction. Milk samples were collected from 161 ewes across 4 sampling periods, measuring productive, compositional, and cheesemaking traits. A linear mixed model was implemented, with fixed effects of breed, sampling period, stage of lactation, parity, and relevant interactions and random effects of animal and residual. Massese ewes showed higher milk yield, resulting in higher component yields and cheese output, whereas Comisana ewes showed higher protein and casein concentrations, leading to better cheesemaking ability. A significant interaction between breed and season revealed breed-specific responses to seasonal variation, with Massese exhibiting more consistent milk production and Comisana showing more pronounced seasonal fluctuations. These findings highlight the importance of considering both breed and season for optimizing milk production and cheesemaking efficiency in local Italian sheep breeds.

## Highlights

• Massese ewes produced more milk, boosting overall cheese output.
• Comisana milk was richer in protein and fat, favoring cheesemaking.
• Milk volume was the main driver of cheese yield.
• Specific breed responses to seasonal variation were observed.

## Graphical Abstract

**Figure:**
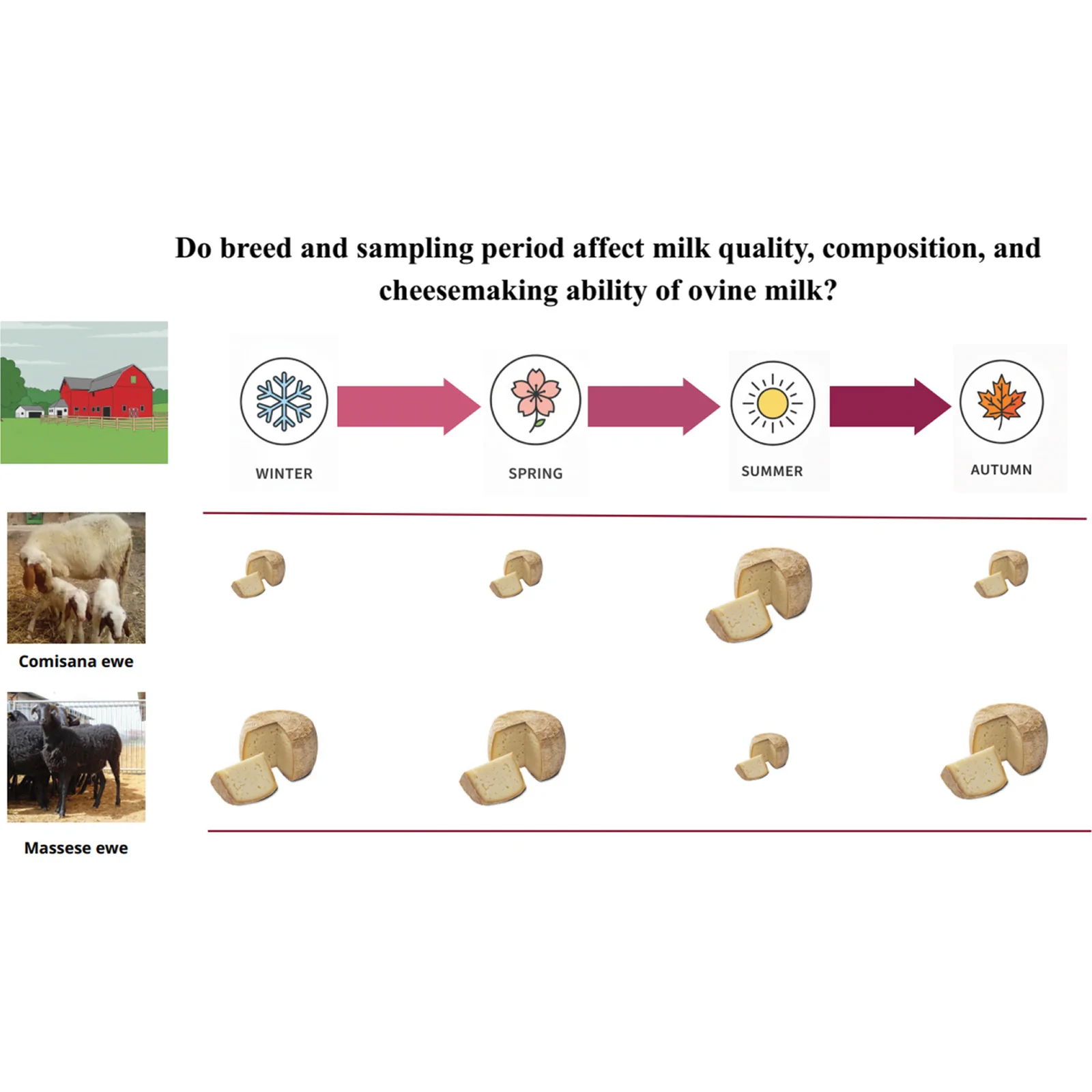
**Summary:** This study examined the effect of breed, sampling period, and their interaction on milk yield, composition, and cheesemaking traits in Comisana and Massese sheep. Results showed that Massese ewes produced higher milk volumes throughout the year, resulting in greater total component yields and cheese output. Comisana ewes showed higher protein and casein concentrations, leading to a higher proportion of curd, but lower milk volume limited overall cheese yield. Interaction between breed and sampling period revealed breed-specific responses: Massese production was more stable across seasons, whereas Comisana showed pronounced seasonal fluctuations. These findings highlight the importance of considering both breed and season when optimizing milk production and cheesemaking efficiency, providing practical insights for managing local Italian sheep breeds.

Small ruminant farming is a longstanding agricultural practice in Mediterranean rural areas, enabling the productive exploitation of marginal lands and supporting rural communities, together with mitigating soil erosion, desertification, and wildfire risk (Boyazoglu and Morand-Fehr, 2001; Vagnoni et al., 2015). Ovine milk differs markedly from bovine milk, with higher protein and fat content, and it is particularly characterized by elevated proportions of medium-chain fatty acids (Park et al., 2007; Li et al., 2022). In several Mediterranean countries, a substantial proportion of ovine milk is processed into traditional dairy products (especially cheeses), reflecting its favorable technological properties, in particular its rapid gelation and curd firming, compared with bovine milk (Pazzola, 2019; Bittante et al., 2022). Italy is a leading producer of ovine milk cheese, including several Protected Designation of Origin products, such as Pecorino Romano and Pecorino Toscano (Biffani et al., 2024). Within dairy production systems, cheese yield (**%CY**), defined as the proportion of raw milk converted into fresh curd, is an important determinant of economic viability and profitability and, together with nutrient recovery in the curd, contributes to defining cheesemaking efficiency (Pazzola et al., 2019). At the individual animal level, milk yield, composition, and technological properties are related to %CY and are influenced by multiple factors, including breed, lactation stage, parity, diet, and seasonal conditions linked to production systems (Cipolat-Gotet et al., 2016a; Li et al., 2022), which complicate the comprehensive characterization of sheep milk used for cheese production (Scintu and Piredda, 2007). Local breeds remain pivotal for sustaining local dairy systems, as they produce milk with distinctive nutraceutical and technological attributes that contribute to the unique qualities of traditional products (Watkins et al., 2021). In Italy, the Comisana and Massese are 2 of the 13 local sheep breeds currently under genetic selection and registered in the National Herd Book managed by the Associazione Nazionale della Pastorizia (**Asso.Na.Pa**; Rome, Italy). The Comisana, whose population consists of ∼2,296 individuals, originates from Sicily and is mainly distributed in the inland areas of central and southern Italy (FAO, 2024). It is characterized by a white coat with a reddish-brown face and semi-lop ears, and is naturally polled. Although traditionally reared in extensive or semi-extensive systems, the Comisana breed shows good adaptability to more intensive production systems, including partially confined housing, mechanical milking, and the use of concentrate-based diets. The breed exhibits a fertility rate of ∼95% and a prolificacy of ∼180%, with an average age at first lambing of 16 mo. The Massese, native to the Apuan Alps in the Tuscany region, is mainly distributed in central Italy (FAO, 2024), with a population consisting of ∼6,735 individuals (FAO, 2024). It exhibits a light gray to black fleece and a typically black face; both sexes are horned, and their strong limbs contribute to enhancing grazing efficiency (Stocco et al., 2025). Massese ewes usually reach their first lambing at ∼15 to 18 mo of age and are characterized by high fertility and prolificacy. Together with their almost continuous estrous activity throughout the year, these traits enable farmers to aim for 3 lambings within a 2-year period, in contrast with the traditional Mediterranean management system, which generally involves a single lambing per year occurring in late spring or early autumn (Todaro et al., 2015). Despite their relevance in local production systems, comparative information on their milk yield, composition, and cheesemaking traits under uniform management conditions remains limited.

The objective of this study was to characterize milk yield, composition, and cheesemaking traits in Comisana and Massese ewes reared under uniform conditions, and to evaluate the effects of breed, sampling period, and their interaction on these groups of phenotypes. Data were collected over one year in a single commercial dairy sheep farm located in the Tuscany region (Siena, Italy). The flock includes ewes of both breeds, managed in a semi-intensive farming system, with indoor housing and daytime, year-round access to pasture. Reproductive management was based on continuous ram exposure, allowing for out-of-season lambing and ensuring milk production throughout the year. The whole milk produced by the animals is processed on-farm into dairy products. During the study period, individual milk from a total of 161 ewes (56 Comisana and 105 Massese, respectively) was sampled in January, May, July, and November of 2024, corresponding to the 4 conventional calendar seasons. Traits investigated included productive (milk yield, in kg/sampling), quality (fat, protein, and casein milk content, expressed as both percentage and yields [kg/sampling]), and cheesemaking ability traits (cheese yield expressed as a percentage [%CY], and cheese yield, expressed in grams [**CYg**]). For each animal, 2 aliquots of individual milk samples were collected during the afternoon milking, immediately stored at 4°C, and analyzed within 24 h of sampling. Information on parity and lambing dates was provided by Asso.Na.Pa. Fat, protein, and casein content were determined by infrared spectrometry using a MilkoScan FT3 (Foss Electric A/S, Hillerød, Denmark) calibrated in accordance with the related reference procedures ISO-IDF (2013), and the values were multiplied by milk yield to determine the corresponding yields (kg/sampling). Cheesemaking ability was assessed on individual samples, reproducing the process on a small-scale laboratory method proposed by Cipolat-Gotet et al. (2016b) using the 9-mL milk assessment. The %CY trait was determined as the ratio between the fresh curd weight (g) and the weight of the milk processed (g) multiplied by 100. The %CY was multiplied by the corresponding milk yield to calculate cheese yield in grams (CYg) for each observation. Because 2 milk samples were collected from each animal at each sampling period, replicate measurements were averaged to obtain a single value per trait per animal per sampling period. Values outside the range of 3 SD were considered outliers and treated as missing values. Stage of lactation was defined as early (earlier than 75 DIM), mid (from 76 to 120 DIM), and late (later than 120 DIM). Parity was classified into 2 levels: primiparous (first lactation) and multiparous (second or subsequent lactations). Observations were balanced across the levels of breed, sampling period, parity, and stage of lactation. Each trait (production, quality, and cheesemaking) was analyzed with a linear mixed model using the lme4 package (Bates et al., 2015) of R (version 4.4.3, R Core Team, 2025). The model had the following form:

*y_lmnop_* = *μ* + *B_l_* + *SP_m_* + *B_l_* × *SP_m_* + *P_n_* + *SOL_o_* + *P_n_* × *SOL_o_* + *Animal_p_* + *e_lmnop_*,

where *y_lmnop_* is the trait under evaluation (fat, protein, and casein content; %CY; and CYg); *µ* is the overall intercept of the model; *B_l_* is the fixed effect of the *l*th breed (*l* = Comisana, Massese); *SP_m_* is the fixed effect of the *m*th period of sampling (*m* = January, May, July, November); *B_l_* × *SP_m_* is the fixed effect of the interaction between the levels of the *l*th effect of breed and the levels of the *m*th effect of period of sampling; *P_n_* is the fixed effect of *n*th parity (*n* = primiparous, multiparous); *SOL_o_* is the fixed effect of the *o*th stage of lactation (*o* = early, medium, late); *P_n_* × *SOL_o_* is the fixed effect of the interaction between the levels of the *n*th effect of parity and the levels of the *o*th effect of stage of lactation; *Animal_p_* is the random effect of the animal, and *e_lmnop_* is the random residual. The native ‘anova’ R function with the Kenward–Roger method was used to test the contribution of the fixed effects. Least squares means were obtained using the emmeans package in R (Lenth, 2025). Pairwise comparisons among sampling periods within breed were then performed, and differences were identified using a compact letter display based on Tukey-adjusted comparisons. Pearson correlation analysis was performed to evaluate the relationships between milk composition traits and cheesemaking parameters. Descriptive statistics for milk yield, milk quality, and cheesemaking traits are reported in Table 1. Average milk yield was 0.35 ± 0.14 kg and 0.39 ± 0.16 kg for Comisana and Massese ewes, respectively, with a coefficient of variation of ∼41% in both breeds. Considering that the values of the present study were obtained from a single milking, average milk yield was consistent with that reported by Stocco et al. (2025), but lower than that reported by Ramirez-Diaz et al. (2025). Fat, protein, and casein contents averaged 7.57% ± 1.33%, 6.04% ± 0.83%, and 4.52% ± 0.62%, respectively, in Comisana milk and 7.41% ± 1.34%, 5.74% ± 0.67%, and 4.33% ± 0.50% in Massese milk. Milk component values for Comisana ewes were higher than those reported by Ramirez-Diaz et al. (2025), probably due to lower milk yield, whereas Massese values were slightly different but comparable with those obtained by Pugliese et al. (1999) and Ramirez-Diaz et al. (2025). Although %CY exhibited greater variability than that reported by Stocco et al. (2025), mean values were comparable across studies. Massese ewes consistently produced more milk, resulting in higher fat, protein, and casein yields, as well as greater CYg. Comisana ewes showed slightly higher protein and casein concentrations, consistent with their higher %CY. Consistently, breed-specific Pearson correlations indicated a positive association between protein content and cheesemaking traits in both breeds. In particular, protein content, expressed as both concentration and yield, was positively correlated with %CY and CYg (r = 0.36 and r = 0.83, respectively, in Comisana; r = 0.33 and r = 0.85 in Massese; *P* < 0.01), confirming the importance of milk protein for curd formation and cheese production. Because %CY is influenced by milk fat and protein contents, which determine the quantity of milk constituents retained in the curd (Pazzola et al., 2019), Comisana milk may offer more favorable characteristics for the cheesemaking process, as also reported by Stocco et al. (2025). However, this compositional advantage did not translate into higher CYg, as the lower milk volume ultimately limited cheese output. This finding suggests that a higher %CY does not necessarily correspond to a higher cheese yield when milk volume is limited.

**Table 1:** Descriptive statistics of productivity, quality, and cheesemaking ability traits per breed

Trait^1^	Descriptive statistic					
	N	Mean	SD	Minimum	Maximum	CV
Comisana						
Productivity						
Milk yield (kg/sampling)	90	0.35	0.14	0.10	0.80	41.13
Quality						
Fat (%)	86	7.57	1.30	4.59	10.40	17.22
Fat yield (kg/sampling)	86	0.03	0.01	0.01	0.06	39.97
Protein (%)	88	6.00	0.83	4.35	7.88	13.91
Protein yield (kg/sampling)	88	0.02	0.01	0.01	0.05	35.93
Casein (%)	88	4.52	0.62	3.26	5.89	13.78
Casein yield (kg/sampling)	88	0.02	0.01	0.01	0.04	36.13
Cheese yield						
%CY	66	25.70	5.11	15.61	43.46	19.87
CYg	66	93.62	31.66	42.11	205.75	33.82
Massese						
Productivity						
Milk yield (kg/sampling)	183	0.39	0.16	0.10	0.90	41.02
Quality						
Fat (%)	178	7.41	1.34	4.28	10.96	18.05
Fat yield (kg/sampling)	178	0.03	0.01	0.01	0.07	42.64
Protein (%)	183	5.70	0.67	4.15	7.83	11.83
Protein yield (kg/sampling)	183	0.02	0.01	0.01	0.05	37.67
Casein (%)	183	4.30	0.50	3.13	5.90	11.70
Casein yield (kg/sampling)	183	0.02	0.01	0.01	0.04	37.92
Cheese yield						
%CY	108	24.01	3.93	15.34	31.86	16.37
CYg	108	109.85	33.41	39.73	209.95	30.41

1 Milk yield = milk from one milking; %CY = curd percentage on the total amount of milk processed; CYg = cheese yield, in grams.

Results of ANOVA are reported in Table 2. The effect of sampling period significantly affected all traits. In particular, fat concentration was higher in January and November (data not shown), likely reflecting both the extensive use of hay during colder months, which increases dietary fiber and stimulates milk fat synthesis (Kaufmann and Hagemeister, 1987), and a concentration effect associated with the reduction in milk yield. A similar concentration effect may explain the higher protein content observed in January and May (data not shown), together with the availability of fresh spring pasture. The concurrent reduction in milk yield and increase in fat and protein content in January were consistent with Sevi et al. (2004). Conversely, milk yield increased in July (Figure 1) likely due to the absence of heat stress, as the barn was equipped with cooling systems, whereas fat, protein, and casein percentages declined (Figure 1), probably as a consequence of dilution effect. Breed differences were significant for protein percentage (*P* = 0.01), casein percentage (*P* = 0.01), and %CY (*P* = 0.01). Stage of lactation had an impact on milk yield (*P* < 0.001), fat percentage (*P* = 0.003), protein percentage (*P* < 0.001), and casein percentage (*P* < 0.001). Parity and its interaction with stage of lactation were generally not significant, except for a minor effect on protein percentage (*P* = 0.03). The breed × sampling period interaction significantly affected all traits, including protein (*P* < 0.001), casein (*P* < 0.001), fat (*P* = 0.009), milk yield (*P* = 0.009), %CY (*P* = 0.02), and CYg (*P* = 0.02). The significant interaction between breed and sampling period indicates breed-specific responses to seasonal changes, as also supported by the trend of the LSM shown in Figure 1. Within-breed comparisons further highlighted these patterns, as indicated by the different letters in Figure 1. Overall, Massese ewes showed a more consistent milk yield throughout the year, resulting in higher overall cheese production, whereas Comisana ewes showed more pronounced seasonal fluctuations. This result may be explained by the greater absorption of thermal radiation in dark-coated animals, which can increase their susceptibility to heat stress compared with animals with lighter coat color (McManus et al., 2020). However, it should be noted that milk yield in the present study was obtained from a single milking rather than a 24-h milk yield; therefore, comparisons with studies reporting daily milk production should be interpreted with caution. Within this context, the observed differences suggest that the 2 breeds may differ in sensitivity to environmental variation, with potential implications for farm management. In particular, breeds showing more stable production patterns under variable environmental conditions could offer advantages in systems characterized by climatic or nutritional variability, whereas those exhibiting stronger seasonal responses may benefit from targeted management strategies aimed at mitigating periods of reduced performance. Because both breeds were evaluated under the same farming conditions, the observed differences reflect breed performance within this specific production context and may vary under alternative production systems.

**Table 2:** Results from ANOVA (*F*-values and significance) for productivity, quality, and cheesemaking ability traits^1^

Trait	SP	B	P	SOL	P × SOL	B × SP
Productivity						
Milk yield (kg/sampling)	8.73***	0.70	1.72	8.31***	1.75	3.88**
Quality						
Fat (%)	23.45***	3.17	1.09	5.72**	1.88	3.91**
Protein (%)	24.64***	6.65*	3.56	14.39***	3.33*	10.37***
Casein (%)	25.08***	6.04*	3.74	14.31***	3.03	10.62***
Cheese yield						
%CY	56.61***	6.32*	2.09	0.46	1.66	3.23*
CYg	5.05**	2.83	2.64	1.74	0.84	3.38*

1 SP = sampling period; B = breed; P = parity; SOL = stage of lactation; milk yield = milk from one milking; %CY = curd percentage on the total amount of milk processed; CYg = cheese yield, in grams.

* *P* < 0.05, **P < 0.01, ***P < 0.001.

**Figure 1:**
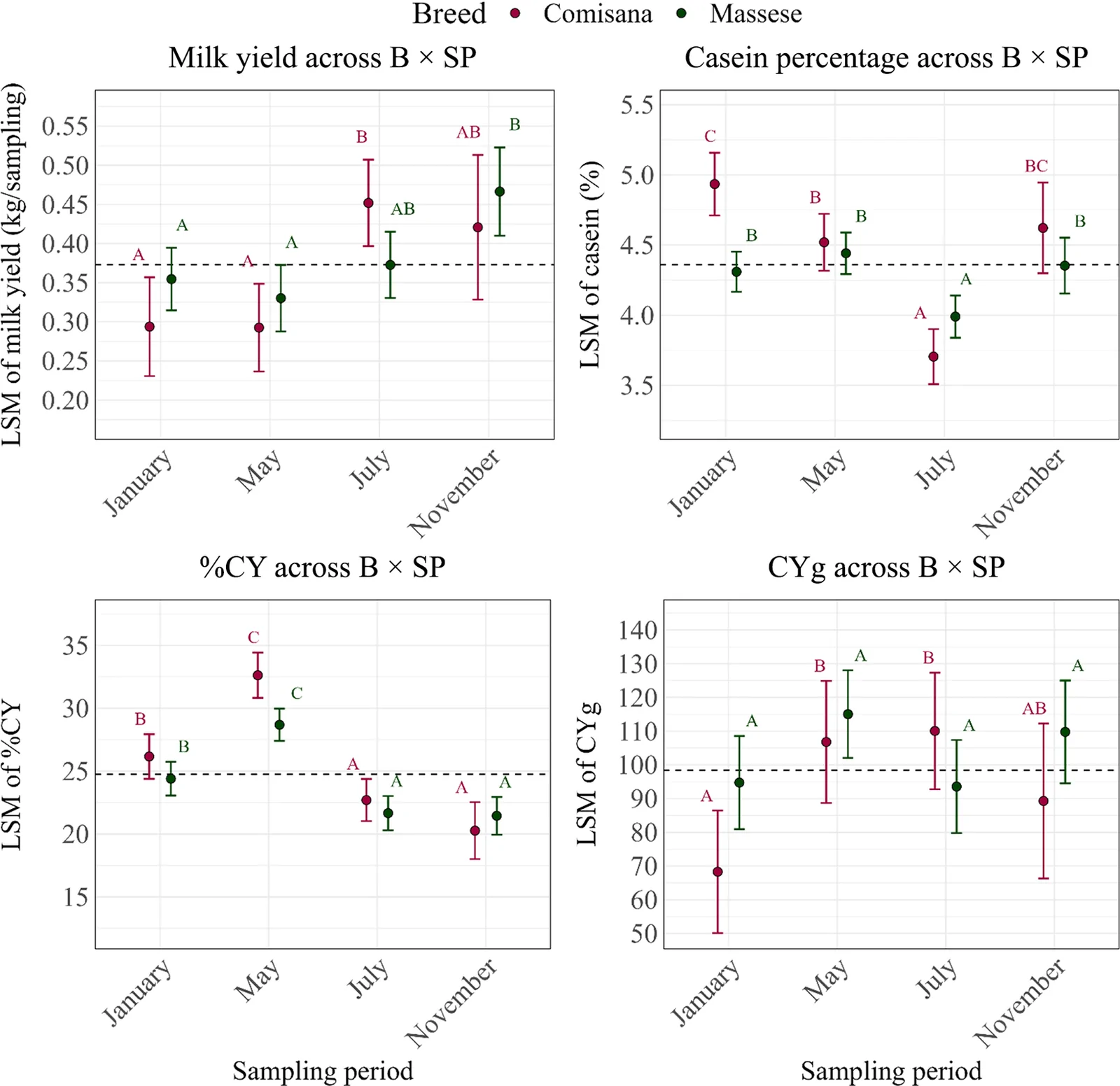
Least squares means of milk yield, casein percentage, %CY, and CYg across the levels of the interaction of breed (B) and sampling period (SP). The dashed line represents the overall mean of the LSM across all B × SP combinations, whereas different letters indicate significant differences among sampling periods within each breed (*P* < 0.05). Error bars represent SE of LSM.

Despite some limitations, including the use of data from a single farm, a single year of observations, and a relatively limited number of animals, this study showed clear breed differences in milk composition and cheesemaking traits, while also highlighting the strong seasonal effects that determine milk yield and quality throughout the year. We acknowledge that litter size might have an impact on milk yield and composition. Because we did not have this information available, this might result in a potential pitfall. The results indicate that milk yield remains the primary driver of cheese output, with compositional advantages playing a secondary role in practical cheesemaking efficiency. The distinct seasonal responses observed between breeds suggest that Massese ewes may provide more consistent cheese production under variable environmental conditions, whereas Comisana ewes may benefit from more targeted nutritional or managerial adjustments during specific periods. From a practical perspective, the different production patterns observed between breeds may also have implications for the composition of bulk milk in multibreed flocks. The more stable milk yield observed in Massese ewes may contribute to maintaining milk supply throughout the year, whereas the slightly higher protein and casein concentrations observed in Comisana milk may improve milk technological quality. These complementary characteristics could represent an advantage for dairy sheep farms processing milk into cheese, helping to balance milk availability and cheesemaking performance across seasons. Overall, these findings offer useful insights for optimizing feeding strategies, improving cheesemaking efficiency, and supporting the sustainable management and valorization of Italian local sheep breeds.
